# Myeloid-Derived microRNAs, miR-223, miR27a, and miR-652, Are Dominant Players in Myeloid Regulation

**DOI:** 10.1155/2014/870267

**Published:** 2014-08-11

**Authors:** Anna B. Gilicze, Zoltán Wiener, Sára Tóth, Edit Buzás, Éva Pállinger, Franco H. Falcone, András Falus

**Affiliations:** ^1^Department of Genetics, Cell- and Immunobiology, Semmelweis University, Nagyvárad tér 4, Budapest 1089, Hungary; ^2^Division of Molecular & Cellular Science, The School of Pharmacy, University Park Science Road, Nottingham NG7 2RD, UK

## Abstract

In the past few years expanding knowledge has been accumulated about the role of microRNAs (miRNAs) not only in hematopoiesis and cancer, but also in inflammatory and infectious diseases. Regarding myeloid cells, our knowledge is relatively insufficient, therefore we intended to collect the available data of miRNA profiles of myeloid cells. In addition to a rather general myeloid regulator miR-223, two other miRNAs seem to be useful subjects in understanding of myeloid miRNA biology: miR-27a and miR-652. We review functions of these three miRNAs and other myeloid miRNAs focusing on their roles in monocytes, neutrophils, eosinophils, basophils and mast cells.

## 1. Introduction

MicroRNAs (miRNAs) are a family of small, evolutionary conserved, and noncoding RNAs that regulate gene expression negatively at the posttranscriptional level—binding complementary sequences within the 3′ untranslated regions of their target mRNA and inducing their degradation or translation inhibition. miRNAs are generated via a specialized pathway that involves the RNase Dicer that produces RNA duplexes of 19–23 nucleotides in length, the mature miRNAs [1].

**Figure 1:**
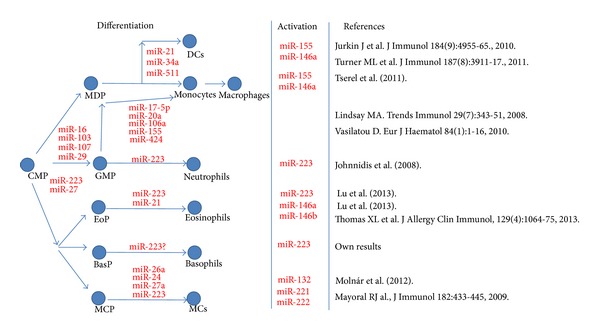
An overview on micro RNA regulation in myeloid development.

miRNAs were first described as factors involved in cell development and differentiation, but later their importance was verified in many other biological processes, such as cell fate determination, apoptosis, signal transduction, and organ development and in various human diseases (Table 1) such as developmental abnormalities, cancer (including haematological malignancies), inflammatory diseases [2, 3], asthma [4], and infection [4]. It is now well established that miRNAs play a role both in the differentiation and the function of peripheral blood cells. Data are rapidly accumulating regarding leukocytes and their miRNA signatures. According to a microarray study from selected immune blood cell populations, there are more miRNAs in myeloid lineages than in lymphoid lineages. Although it means a relatively low number of few miRNAs, they may exert significant secondary effects on the transcription of other genes by directly repressing the expression of genes involved in translational regulation [5] (Figure 1).

Innate immunity provides the first line of protection upon pathogen invasion, and it mainly involves cells of myeloid lineages. These cells are capable of mediating rapid clearance of invading pathogens by phagocytosis, and they are rapidly recruited to local tissues at the site of infection via various chemokine receptors, leading to a complex sequence of inflammatory responses, including vasodilatation and increased vascular permeability [6]. Abnormalities in the development of myeloid cells may cause an abnormal increase in mature myeloid or blast cells resulting in chronic or acute myelogenous leukemia. Acute myeloid leukemia (AML) is divided into 8 subtypes based on abnormal cell types and degree of maturity [7]. Myeloid cells can facilitate angiogenesis and tumor growth by both VEGF-dependent and VEGF-independent manners, and moreover, by their capability to induce immunosuppressive effects [8].

There are a few miRNA microarray studies investigating miRNA patterns of blood cells. Regarding miRNA pattern of myeloid cells, there are some information about neutrophils and monocytes, a few about myeloid dendritic cells (DCs), and very few about eosinophils and mast cells. The information about basophils is still lacking.

There are data on the miRNA profile of 9 types of leukocytes (including neutrophils, eosinophils, monocytes, and mDCs) by array. The authors found that nine miRNAs were specific to myeloid lineage cells: miR-223, miR-143, miR-145, miR-25, miR-27, miR-425, miR-17, miR-652, and miR-191. Also an mRNA array was performed on the same set of samples in order to determine if cell-type specific miRNAs downregulated the expression levels of their target genes. It was found that C-Abl oncogene 2, nonreceptor tyrosine kinase, (ABL2, it regulates cytoskeleton remodelling during cell differentiation, cell division and cell adhesion) was targeted by the largest number of myeloid specific miRNAs. These miRNAs may promote differentiation and granulopoiesis by repressing ABL2 levels, disrupting maintenance of the undifferentiated myeloid precursor state [5]. In another miRNA microarray study, the role of miRNAs was investigated on gene expression of neutrophils after physical exercise. Many of the exercise-affected miRNAs in neutrophils are known to regulate genes involved in immune processes and apoptosis including miR-17, miR-18a, and miR-20a (all part of the miR-17-92 cluster, which had decreased expression level after exercise). In case of miR-223, the authors found elevated expression immediately after exercise. The authors found three major pathways in neutrophils in which both exercise-influenced miRNAs and mRNAs are involved: the ubiquitin-mediated proteolysis pathway, the Jak-STAT—and the Hedgehog signaling pathways. Each of these pathways plays a role in key mechanisms of inflammation [25]. A further neutrophil study, where miRNA expression of freshly isolated human neutrophils was analysed by microarray, found that the most abundant miRNA in all samples at any time point was miR-223 [26]. In the case of mast cells (MCs), there is only one murine miRNA microarray study that analysed miRNA patterns during differentiation and activation of bone-marrow derived mast cells. MiR-132 showed the highest upregulation upon activation by IgE/antigen, which regulates heparin-binding epidermal-like growth factor (HB-EGF) expression at the translational level. Expression of miR-132 was confirmed in human cord-blood derived mast cells [27]. In a study by Tserel et al. the miRNA pattern of monocytes, DCs, and macrophages was analysed by microarray, where miR-511, miR-99b, and miR-212 were detected as the three most highly upregulated miRNAs in both DCs and macrophages. According to the miRNA patterns, monocytes are more similar to macrophages than dendritic cells, as shown by hierarchical clustering analysis. MiR-146a and miR-132 were detected as differentially regulated miRNAs during cell differentiation [28].

After collecting data about miRNA patterns of myeloid cells based on miRNA microarray analysis from above studies, there were 2 miRNAs (miR-27a and miR-652) which seemed to be common in myeloid cells as they are expressed by at least 4 of the myeloid cells in our focus (Table 2). In the case of mast cells, only murine data are available, and we presented miRNAs which were significantly upregulated during MC differentiation or could be activated by Fc*ɛ*RI crosslinking in the case of MCs in Table 2. MiR-223 is significantly downregulated during MC differentiation. Regarding miR-27a, in an earlier study its expression was also observed in mature murine bone marrow-derived MCs [29]. It is well known that miR-223 has a key role in myeloid development and function (Figure 1), but in the case of miR-27a and miR-652 there is no evidence for this, and our knowledge about the role of these miRNAs in myeloid cells is rather poor. Our findings presented in this paper suggest that basophils also express miR-223. MiRNAs have been demonstrated to be present in serum or plasma, where they are resistant to RNase digestion, and their expression is consistent among individuals. There are specific expression patterns of serum miRNAs in certain diseases, therefore serum or plasma miRNAs can be used as fingerprints of various diseases [30].

After reviewing miR-223, miR-27a, and miR-652, we detail some other miRNAs, which are known to be important players in myeloid biology.

## 2. A Dominant Player, miR-223

miR-223 is an essential and central modulator of myeloid differentiation and may control granulocytic differentiation in humans [31].

Using the miR-223 gene targeted (KO) murine model, it was shown that miR-223 is a negative regulator of granulocytic differentiation and activation in neutrophils. MiR-223 null (miR-223 −/Y chimaeric) mice had increased numbers of granulocyte progenitors in the bone marrow and showed a hypermature and hypersensitive circulating neutrophil-like phenotype (nuclear hypersegmentation and blebbing), with an aberrant pattern of lineage-specific marker expression. These mice also had inflammatory lung lesions and demonstrated enhanced tissue destruction following bacterial endotoxin challenge [32]. In an miRNA microarray study it was shown that miR-223 is the most abundant and likely the most important miRNA in human peripheral blood neutrophil granulocytes [26].

In the case of eosinophils, miR-223 ablation was shown to cause increased proliferation of eosinophil progenitors, delayed differentiation and upregulation of miR-223 targeted gene insulin-like growth factor receptor (IGF1R) in them [33].

**Figure 2:**
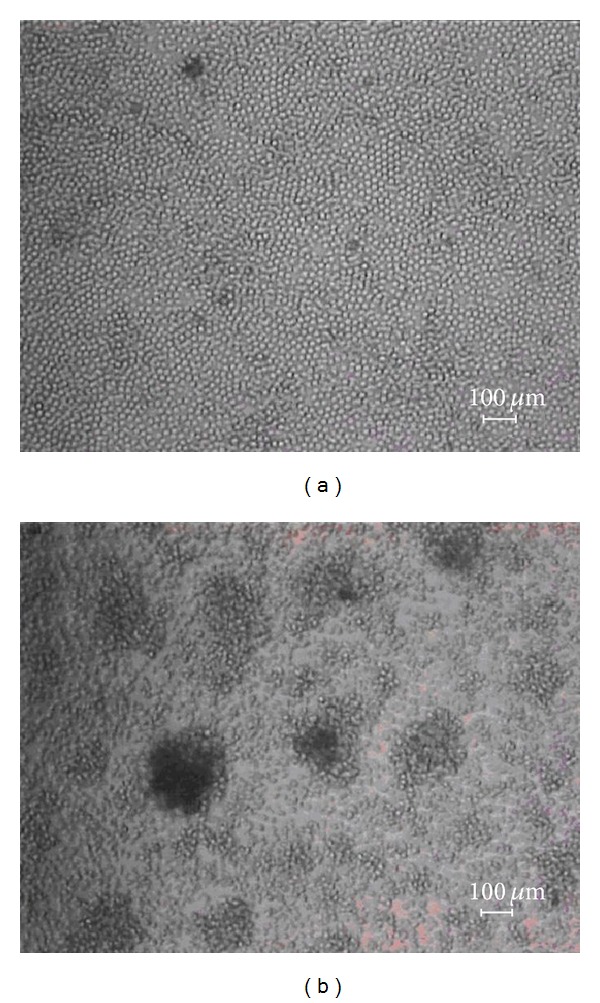
Basophils purified from human peripheral blood (>99% pure, data not shown) before (a) and (b) after treatment by 10 ng/mL hIL-3 for 100 minutes.

**Figure 3:**
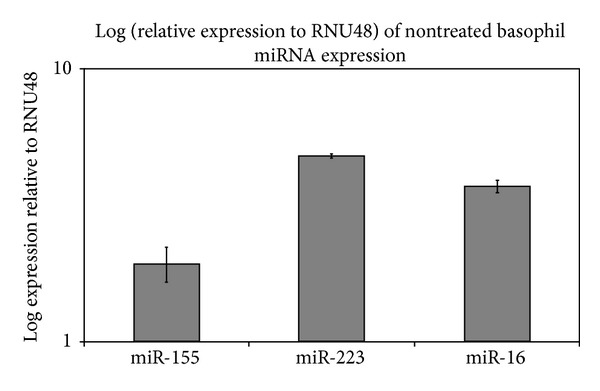
Expressions of miR-155, miR-16, and miR-223 in basophils of healthy untreated human subjects (standardized to RNU48-GATGACCCCAGGTAACTCTGAGTGTGTCGCTGATGCCATCACCGCAGCGCTCTGACC) (Life Technologies), *n* = 17.

In basophils purified from human peripheral blood (>99% pure, data not shown), as shown in Figures 2(a) and 2(b), our data show that after 100 minutes of cell treatment by human interleukin-3 (hIL-3), a pronounced clustering of cells can be observed. Basophils, cultured in the presence of hIL-3, formed clusters, while control cells remained evenly distributed in the culture. As shown in Figure 3 (representative sample), both miR-16 and miR-223 were highly expressed, while miR-155 expression was lower albeit pronounced in our healthy untreated human basophil samples.  Table 3 shows the fold changes of hIL-3 treated samples. All of the investigated miRNAs showed reduced expression upon hIL-3 treatment. MiR-233 expression showed the most pronounced decrease in expression, followed by miR-155 and miR-16.

It seems that the importance of miR-223 is not confined to granulocytic differentiation. In monocyte/macrophage differentiation, miR-233 modulates NF-*κ*B activation and targets IKK-*α* kinase, a member of NF-*κ*B pathway. During monocyte/macrophage differentiation, miR-223, miR-16, and miR-15a are all decreased, and IKK-*α* is increased, which then contributes to relB/p52 production and repression of both canonical and noncanonical NF-*κ*B pathways. Therefore, it prevents macrophages from overactivation but possibly sensitizes them for future NF-*κ*B signalling events. It is suggested that the decrease in certain miRNAs during differentiation leads to a condition where macrophages can adequately respond to infection, but where additional tissue damages are prevented [34].

MiR-223 antagonises angiogenesis, as its overexpression inhibits both the vascular endothelia growth factor- (VEGF-) and basic fibroblast growth factor- (bFGF-) induced phosphorylation of the respective receptors and activation of protein kinase B (PKB/Akt). This phenomenon correlates with downregulation of miR-223 target *β*1-integrin [35].

MiR-223 targets an aniridia type II protein (AN2/PAX6) or oculorhombin, a transcription factor present during embryonic development, and acts as a tumor suppressor in glioblastoma, as well. MiR-223 overexpression and AN2/PAX6 knockdown caused growth and invasion of glioblastoma cells and increased matrix metalloproteinases, MMP-2, MMP-9, and VEGF-A expression [4]. In breast cancer, miR-223 suppresses the proliferation and invasion of cancer cells by targeting cytoplasmic activation/proliferation-associated protein 1 (Caprin-1) [10].

MiR-223 expression rises incrementally during successive stages of granulocytic differentiation. In this process miR-223 targets the transcription factor nuclear factor 1 A (NFI-A), which is required for granulocyte/neutrophil differentiation and which competes with myeloid specific factor C/EBP*α* for binding miR-223 promoter. These miRNAs induce a regulatory loop in myeloid progenitor development (commitment) and differentiation, where NFI-A is downregulated by miR-223 and miR-223 is upregulated by CCAAT/enhancer binding protein (C/EBP*α*). The increase in miR-223 expression results from the displacement of the inhibitor NFI-A by C/EBP*α* [31, 36]. Based on other data, the situation seems to be even more complex. MiR-223 inhibits the transcription factor myocyte enhancer factor-*2* (Mef2c), which is involved in promoting myeloid progenitor differentiation and which seems to be responsible for the changes of the amount and the phenotype of neutrophils in gene targeted, miR-223-deficient (KO) mice mentioned earlier [32]. Beside C/EBP, myeloid transcription factor PU.1, a key regulator of haematopoietic differentiation also induces miR-223 expression, while erythroid transcription factor globin transcription factor 1 (GATA1) represses it [37]. C/EBP*α* downregulates cell-cycle regulator E2F1 protein during granulopoiesis, and E2F1 inhibits miR-223 transcription, while miR-223 targets E2F1 and blocks cell cycle progression in AML cells, forming a negative autoregulatory loop [13]. In AML, it was shown that C/EBP*α* contributes directly to the development of the disease [38].

It was also shown that miR-223 is downregulated in human AML [13]. Runt-related transcription factor 1 (RUNX1) mutations have been associated with poor clinical outcome in younger patients with cytogenetically normal AML. In RUNX1-mutated AML patients, C/EBP*α* is downregulated [39]. C/EBP*α* mediated upregulation of miR-223 could be a possible explanation of the phenomenon that miR-223 is largely suppressed in cells from AML patients [14].

Interestingly, in a recent study by Filkova et al. [3], it was suggested that by monitoring miR-16 and miR-223 levels, the disease outcome in early phase rheumatoid arthritis (ERA) patients could be predicted. The authors found lower circulating miR-16, miR-155, and miR-146 levels in ERA and healthy controls' samples than in those of patients with rheumatoid arthritis (RA). Furthermore, a relationship was shown between the miR-223 level and peripheral lymphocyte number changes in ERA patients. Moreover, it seemed that a higher circulating miR-223 baseline level could signify a better improvement in disease activity in ERA after therapeutic intervention [3].

In a murine model of asthma, a miRNA microarray analysis had been used from allergen-exposed lung at 3 time-points (short-, intermediate- and long term) of allergen exposure. It was found that miR-223 was significantly upregulated at short-time and was downregulated at intermediate-time of aerosol exposure. Moreover, mRNA potentially targeted by miR-223 (Arid4b, Il-6, and Lpin2) has undergone a downregulation at short-term exposure [15].

MiR-223 is abundantly expressed during active tuberculosis (TB) in humans as it was shown by Dorhoi et al., [17] based on a microarray from active pulmonary TB patients and latently infected healthy individuals. The authors found that similar to human TB, miR-223 was upregulated in* Mycobacterium tuberculosis* infected mice. Moreover, miR-223 deletion rendered resistant mice susceptible to TB, and it seemed that miR-223 regulated NF*κ*B activity in macrophages and cytokine release in myeloid cells during TB. As it was shown by another microarray experiment from lung tissue on days 14 and 21 during Mtb infection, lung influx of innate cells during TB was modulated by miR-223 through the regulation of chemoattractants. Cxcl2, Ccl3, and IL-6 were identified as new targets of miR-223. Mortality in infected miR-223 KO animals was 80–100%, while it was only 10% in “wild-type” littermates. Neutrophil depletion prolonged survival of the animals; therefore the major cause of morbidity in the absence of miR-223 was considered to be neutrophilic inflammation and subsequent tissue destruction. It was suggested that the miR-223 targeted chemokines CXCL2 and CCL3 and the proinflammatory cytokine IL-6 played an important role in these processes. In the long run, miR-223 induced negative feedback control of neutrophil chemotaxis [17].

Despite the general assumption of miRNA duplexes, both arms of miR-223 duplex have an active role, as it was shown by Kuchenbaueret al. The authors found that miR-223* had a regulatory role in myeloid progenitor cells. In a murine miR-223 KO model, where the seed region of the miR-223 arm was inactivated, the colony-forming cell output was significantly reduced. It was also shown by target predictions and a miR-223 KO-miR-223 WT microarray that miR-223 and miR-223* both target insulin-like growth factor (IGF1r). Therefore they might have cooperative role on IGF1R/PI3 K axis, a key pathway for developmental and malignant processes. The authors found that miR-223* had a higher expression in healthy donors than in AML patients, and a superior overall survival was significantly associated with the high miR-223* expression level [40].

Beside miR-223, two other miRNAs are included in Table 2 as miRNAs, which are present in 4 out of 6 types of myeloid cells: miR-27a and miR-652. Likely, these three miRNAs could be key modulators in myeloid cells, as well.

### 2.1. miR-27a

miR-27a plays a role in granulocytic differentiation as it was shown that the amount of pri-miR-27a in myeloblasts was low, while it was high in granulocytes. Moreover, it targeted the 3′ UTR of RUNX1 transcription factor, a master regulator in hematopoietic development, and miR-27a inhibited the Runx1 protein during differentiation of myeloblasts [41]. It is possible that the miR-27a-Runx1 system involves a feedback loop [41, 42]. Interestingly, a microarray analysis of RUNX1 mutation suggested the importance of miR-223 [39]. It was shown in a murine asthma model (in the case of a long-term aerosol exposure) that miR-27a was significantly downregulated [15].

MiR-27a promotes angiogenesis [11] and can alter the activity of NF-*κ*B, mitogen-activated protein kinases (MAPKs) p38, c-Jun N-terminal kinase (JNK), and extracellular-signal-regulated kinase (ERK) and therefore influence the production of certain proinflammatory cytokines [43].

### 2.2. miR-652

Our knowledge on miR-652 is rather poor. It is specifically regulated in serum of liver disease and is downregulated in circulating monocytes isolated from patients compared to controls. As it was shown that stimulation of monocytic U937 cells with bacterial lipopolysaccharide (LPS) led to a significant downregulation of miR-652, therefore it seems that this miRNA is involved in the mediation of inflammatory signals in immune cells [18].

## 3. Further Myeloid miRNAs with Well Characterized Effects

### 3.1. miR-155

MiR-155 plays a critical role during hematopoiesis and regulates lymphocyte homeostasis and tolerance [44]. In a recent study about miRNA expression profiles in leukocytes, it was observed that miR-155 was specifically expressed in lymphoid lineage cells but it was downregulated in neutrophils, eosinophils, and mDCs [5]. On the other hand, it was shown that a sustained expression of miR-155 in hematopoietic stem cells could increase immature granulocyte numbers* in vivo* [19]. MiR-155 was shown to be required for normal immune function, as gene targeted, miR-155-deficient mice were highly susceptible to* Salmonella typhimurium* infection. Rodriguezet al. [45] also suggested that IL-4 promoter binding of c-Maf transcription factor can be inhibited by loss-of-miR-155. This raises the intriguing possibility that miRNAs may have an important effect even on Th2-related humoral immune responses.

MiR-155 is the most commonly overexpressed miRNA in various malignancies. It is related to cancers of breast, lung, liver, and the lymphatic system; therefore agents that effectively block miR-155 may have an outstanding importance in cancer therapy [46].

According to a murine asthma model, miR-155 is significantly upregulated at intermediate aerosol exposure [15]. It is also important to note that the chromosomal region in which the miR-155 gene is located (chromosome 21q21) has been previously shown to be associated with increased asthma susceptibility in Hutterites in South Dakota, a founder population of European ancestry [47].

In recent studies it has been reported that miR-155 is able to modulate myeloid cells in some pathological conditions related to the central nervous system. MiR-155 expression is significantly increased in circulating CD14^+^ monocytes of multiple sclerosis (MS) patients compared to healthy controls. Transfection of miR-155 increased tumor necrosis factor (TNF)*α*, IL-6, CD80, and CCR7 expression, while the expression of the miR-155 targeted gene, suppressor of cytokine signaling protein (SOCS1) was decreased in human microglia. On the other hand, interferon (IFN)*γ* was significantly increased in supernatants derived from CD4^+^ T cells cocultured with miR-155-transfected monocyte-derived macrophages [20].

### 3.2. miR-146a

LPS-induced elevation of miR-146a, miR-155, and miR-132 was observed in a microarray study of the monocytic leukemia cell line THP-1 after LPS challenge. Therefore, miR-146a seemed to be a negative regulator of inflammation. Toll-like receptors (TLRs) that recognize bacterial constituents (TLR2, TLR4, and TLR5), could trigger miR-146a induction, while TLRs that recognize viral nucleic acids (TLR3, TLR7, and TLR9) had little effect on miR-146a expression [48].

In the absence of miR-146a, hematopoietic stem cell homeostasis is disrupted under aging and periodic bacterial encounters, as indicated by declines of hematopoietic stem cell number and quality, dysregulated hematopoietic stem, and progenitor cell proliferation and differentiation. Chronically, it can lead to myeloproliferative disease. The molecular pathway miR-146a/TRAF6/NF-*κ*B/IL-6 seems to be involved in these processes [49].

Rusca et al. [50] identified a new molecular network that comprises specifically of NF*κ*B1 and miR-146a. The authors have shown that the same activation-induced miRNA can be similarly regulated in different cell types: in mast cells it contributes to the regulation of cell homeostasis and survival, while in T lymphocytes it modulates T cell memory function.

MiR-146a has a role in inflammatory diseases such as psoriasis and rheumatoid arthritis and in cancers (such as papillary thyroid carcinoma, cervical cancer, breast cancer, pancreatic cancer, and prostate cancer) [51]. MiR-146a is upregulated in RA [9]; it is downregulated in systemic lupus erythematosus (SLE) where it is a negative regulator of IFN*γ* pathway [21].

Elevated miR-146a expression was observed in circulating CD14^+^ cells from untreated MS patients [20]. MiR-146a, miR-125b, miR-9, and miR-155 are upregulated in Alzheimer's disease in which this relatively small number of miRNAs may impact several key pathological features [22].

The multilevel effect of the micro-RNA networks is convincingly demonstrated by miR-146A since the complexity of miRNA and transcription factors are illustrated by the fact that not only the inhibition but also the overexpression of miR-146a leads to an inflammatory phenotype.

### 3.3. miR-132

MiR-132 is expressed in mDCs [28] and in murine MCs, and it targets HB-EGF [27].

In human inflammatory bowel disease, miR-132 was found to be a modulator of cholinergic signalling [24]. In MS, miR-132 expression was significantly decreased in circulating CD14^+^ monocytes [20]. The miR-132/miR-212/PTEN/FOXO3a signaling pathway contributes to neuronal apoptosis in Alzheimer's disease [23].

### 3.4. miR-21

miR-21 is a regulator of eosinophil progenitor growth. MiR21+/+ and miR-21−/− bone-marrow derived eosinophils were morphologically indistinguishable from each other, but miR21−/− cells had decreased growth capacity with an increased level of apoptosis* in vitro*. A whole genome microarray study of wild type- and gene targeted cells identified regulation of cell growth, cell cycle, and immune response as the pathways were affected most significantly by miR-21 [52]. MiR-21 is upregulated in allergic airway inflammation [16]. MiR-21 suppresses TLR2 signaling, and IL-13 can upregulate miR-21 expression in human airway epithelial cells [53]. The upregulation of miR-21, miR-142-3p, miR-142-5p, and miR-223 was observed both in human and mouse allergic contact dermatitis [54]. Esophageal miRNA expression profile was investigated by microarray from human esophageal tissues of eosinophilic esophagitis (EoE) patients and controls, where the most upregulated miRNAs were miR-21 and miR-223, and the most downregulated one was miR-375. In the plasma of EoE patients, miR-146a, miR-146b, and miR-223 were upregulated [55].

MiR-21 has a dual role in CD4^+^ T cells: activation-induced miR-21 supports survival of memory T cells, while modulation of the potential homing by downregulation of CCR7 protein expression was identified in active naive T cells [56]. One may consider that* in vivo* silencing of miR-21 reversed cardinal manifestations of autoimmunity in SLE and other diseases [12] suggesting that miRNAs have the potential to become an effective therapeutic target in the future.

In a recent study serum level of miR-21 proved to be a prognostic marker for colorectal cancer as serum miR-21 level correlated with the recurrence and mortality of colorectal cancer patients [57]. In a further meta-analysis study earlier results regarding circulating miR-21 level in cancer were generalized [58].

## 4. Conclusions and Future Perspectives

By now, several studies certified that abnormal miRNA expression is a common feature of various human diseases, and that the number of diseases in which the role of miRNAs has been confirmed, increases continuously. In some cancer types certain circulating miRNAs were identified as potential prognostic markers.

In order to find out how inflammation-induced tumorigenesis works and in order to determine new therapeutic targets, identification and further investigation of key miRNAs involved in inflammation and cancer, could be an expedient strategy. In the case of miR-155 it was reported that miR-155 and miR-155-related proinflammatory environment have mutatory-activity and play a role in inflammation-induced cancers [59]. MiR-146a could be one of the missing links between inflammation and tumorigenesis as suggested by an increasing number of publications that show its importance both in inflammatory diseases and tumors.

Myeloid lineage development and differentiation pathways are rather complex, as miRNAs and transcriptional factors form a complex network, that is, lineage-specific negative and positive regulatory loops. By these loops miRNAs are able to regulate normal hematopoietic lineage development. Moreover, the imbalance or disturbance can evoke diseases by aberrant expression [36].

There is still a limited set of information about miRNA patterns and networks of monocytes, mast cells, eosinophils, and basophils in the literature, while there is growing evidence that inflammation and infections can influence malignancies. Better knowledge of this area of myeloid biology and pathophysiology would be cordially suggested.

In this review we intended to overview information on myeloid miRNAs based on available microarray analysis investigating miRNA pattern of myeloid cells. We focused on monocytes, neutrophils, eosinophils, basophils, and mast cells. There are very few data about myeloid DCs in this review, as DCs are rather different regarding their miRNA expression. We also did not discuss the miRNA regulation of newly identified myeloid-derived suppressor cells.

We could reconfirm the key importance of miR-223 in myeloid cells and provide novel information about miR-223 expression in basophils.

Even though there are only a few miRNA microarray studies of myeloid cells in the literature, based on them it seems that beside the myeloid key miRNA miR-223, miR-27a, and miR-652 and some others could be also interesting regulators with the potential to become therapeutic targets in myeloid pathology, as well.

## Figures and Tables

**Table 1:** Dominant miRNAs in human diseases.

miRNA	Disease	Reference
miR-223, miR-16	rheumatoid arthritis	[3]
miR-146	rheumatoid arthritis	[9]
miR-223	glioblastoma	[4]
miR-223	breast cancer	[10]
miR-27	breast cancer	[11]
miR-21	colorectal cancer	[12]
miR-223	acut myeloid leukemia	[13, 14]
miR-146b, -223, -29b, -29c, -483, -574-5p, -672 and -690	chronic asthma	[15]
miR-21	allergic airway inflammation	[16]
miR-223	tuberculosis	[17]
miR-571 miR-652	liver cirrhosis	[18]
miR-155	many myeloproliferative disorders	[19]
miR-155	multiple sclerosis	[20]
mi-R146A	systemic lupus erythematosus	[21]
miR-7	Alzheimer disease	[22]
miR-132, miR-212	Alzheimer disease	[23]
miR-132	inflammatory bowel diseases	[24]

**Table 2:** Published miRNAs of myeloid cells.

	Mo	Neu	Eo	muc. MCs	DCs
mir-17	h [5]	h [26]			
mir-21		h [26]		m [27]	
mir-25		h [5], h [26]	h [5]		
mir-24-1∗				m [27]	
mir-24-2∗				m [27]	
mir-27a	h [5]	h [5], h [26]	h [5]	m [27]	
mir-34a		h [26]		m [27]	h [28]
mir-93	h [5]	h [5], h [26]	h [5]		
mir-99a					h [28]
mir-99b					h [28]
mir-106a	h [5]				
mir-106b	h [5]	h [5], h [26]	h [5]		
mir-125a-5p		h [5]			h [28]
mir-125a-3p				m [27]	
mir-125b					h [28]
mir-132				m [27]	h [28]
mir-143		h [5]			
mir-145		h [5]			
mir-146a					h [28]
mir-155					h [28]
mir-191	h [5]	h [5]	h [5]		
mir-193b				m [27]	h [28]
mir-212					h [28]
mir-223	h [5]	h [5], h [26]	h [5]		
mir-362-5p	h [5]				h [5]
mir-362-3p				m [27]	
mir-378	h [5]				
mir-422a	h [5]				
mir-425	h [5]	h [5], h [26]	h [5]		
mir-494				m [27]	
mir-500-star	h [5]			m [27]	h [5]
mir-511					h [28]
mir-532-5p	h [5]			m [27]	h [5]
mir-652	h [5]	h [5]	h [5]	m [27]	
mir-720		h [26]			
mir-935			h [5]		

Mo: monocyte, Neu: neutrophil, Eo: eosinophil, Muc MC: mucosal mast cell, DC: dendritic cell.

h: human, m: murine.

For references: see numbers in brackets.

**Table 3:** The effect (fold change) of hIL-3 on miRNA expression in human basophils.

miRNA	untreated	IL-3^§^	*P*
miR-155	1,37	1,084	<0.05
miR-223	4,02	3,47	<0.01
miR-16	3,17	3,01	NS

^§^10 ng/mL hIL-3, 100 min, *n* = 15.

NS: Non significant.

## References

[1] Bartel DP (2004). MicroRNAs: genomics, biogenesis, mechanism, and function. *Cell*.

[2] Sonkoly E, Stahle M, Pivarcsi A (2008). MicroRNAs and immunity: novel players in the regulation of normal immune function and inflammation. *Seminars in Cancer Biology*.

[3] Filkova M, Aradi B, Senolt L (2013). Association of circulating miR-223 and miR-16 with disease activity in patients with early rheumatoid arthritis. *Annals of the Rheumatic Diseases*.

[4] Huang BS, Luo QZ, Han Y, Li XB, Cao LJ, Wu LX (2013). microRNA-223 promotes the growth and invasion of glioblastoma cells by targeting tumor suppressor PAX6. *Oncology Reports*.

[5] Allantaz F, Cheng DT, Bergauer T (2012). Expression profiling of human immune cell subsets identifies miRNA-mRNA regulatory relationships correlated with cell type specific expression. *PLoS ONE*.

[6] Kawamoto H, Minato N (2004). Myeloid cells. *The International Journal of Biochemistry & Cell Biology*.

[7] Tefferi A, Thiele J, Vardiman JW (2009). The 2008 World Health Organization classification system for myeloproliferative neoplasms: order out of chaos. *Cancer*.

[8] Shojaei F, Zhong C, Wu X, Yu L, Ferrara N (2008). Role of myeloid cells in tumor angiogenesis and growth. *Trends in Cell Biology*.

[9] Nakasa T, Miyaki S, Okubo A (2008). Expression of MicroRNA-146 in rheumatoid arthritis synovial tissue. *Arthritis & Rheumatism*.

[10] Gong B, Hu H, Chen J (2013). Caprin-1 is a novel microRNA-223 target for regulating the proliferation and invasion of human breast cancer cells. *Biomedicine & Pharmacotherapy*.

[11] Tang W, Yu F, Yao H (2013). miR-27a regulates endothelial differentiation of breast cancer stem like cells. *Oncogene*.

[12] Garchow BG, Encinas OB, Leung YT (2011). Silencing of microR6-21 in vivo ameliorates autoimmune splenomegaly in lupus mice. *EMBO Molecular Medicine*.

[13] Pulikkan JA, Dengler V, Peramangalam PS (2010). Cell-cycle regulator E2F1 and microRNA-223 comprise an autoregulatory negative feedback loop in acute myeloid leukemia. *Blood*.

[14] Eyholzer M, Schmid S, Schardt JA, Haefliger S, Mueller BU, Pabst T (2010). Complexity of miR-223 regulation by CEBPA in human AML. *Leukemia Research*.

[15] Garbacki N, di Valentin E, Huynh-Thu VA (2011). MicroRNAs profiling in murine models of acute and chronic asthma: a relationship with mRNAs targets. *PLoS ONE*.

[16] Lu TX, Munitz A, Rothenberg ME (2009). MicroRNA-21 is up-regulated in allergic airway inflammation and regulates IL-12p35 expression. *Journal of Immunology*.

[17] Dorhoi A, Iannaccone M, Farinacci M (2013). MicroRNA-223 controls susceptibility to tuberculosis by regulating lung neutrophil recruitment. *The Journal of Clinical Investigation*.

[18] Roderburg C, Mollnow T, Bongaerts B (2012). Micro-RNA profiling in human serum reveals compartment-specific roles of miR-571 and miR-652 in liver cirrhosis. *PLoS ONE*.

[19] O'Connell RM, Rao DS, Chaudhuri AA (2008). Sustained expression of microRNA-155 in hematopoietic stem cells causes a myeloproliferative disorder. *The Journal of Experimental Medicine*.

[20] Moore CS, Rao VT, Durafourt BA (2013). miR-155 as a multiple sclerosis-relevant regulator of myeloid cell polarization. *Annals of Neurology*.

[21] Tang Y, Luo X, Cui H (2009). MicroRNA-146a contributes to abnormal activation of the type I interferon pathway in human lupus by targeting the key signaling proteins. *Arthritis and Rheumatism*.

[22] Lukiw WJ, Andreeva TV, Grigorenko AP, Rogaev EI (2013). Studying micro RNA function and dysfunction in Alzheimer's disease. *Frontiers in Genetics*.

[23] Wong HA, Veremeyko T, Patel N (2013). De-repression of FOXO3a death axis by microRNA-132 and -212 causes neuronal apoptosis in Alzheimer’s disease. *Human Molecular Genetics*.

[24] Maharshak N, Shenhar-Tsarfaty S, Aroyo N (2013). MicroRNA-132 modulates cholinergic signaling and inflammation in human inflammatory bowel disease. *Inflammatory Bowel Diseases*.

[25] Radom-Aizik S, Zaldivar F, Oliver S, Galassetti P, Cooper DM (2010). Evidence for microRNA involvement in exercise-associated neutrophil gene expression changes. *Journal of Applied Physiology*.

[26] Ward JR, Heath PR, Catto JW, Whyte MKB, Milo M, Renshaw SA (2011). Regulation of neutrophil senescence by microRNAs. *PLoS ONE*.

[27] Molnár V, É Rsek B, Wiener Z (2012). MicroRNA-132 targets HB-EGF upon IgE-mediated activation in murine and human mast cells. *Cellular and Molecular Life Sciences*.

[28] Tserel L, Runnel T, Kisand K (2011). MicroRNA expression profiles of human blood monocyte-derived dendritic cells and macrophages reveal miR-511 as putative positive regulator of toll-like receptor 4. *Journal of Biological Chemistry*.

[29] Monticelli S, Ansel KM, Xiao C (2005). MicroRNA profiling of the murine hematopoietic system. *Genome Biology*.

[30] Chen X, Ba Y, Ma L (2008). Characterization of microRNAs in serum: a novel class of biomarkers for diagnosis of cancer and other diseases. *Cell Research*.

[31] Fazi F, Rosa A, Fatica A (2005). A minicircuitry comprised of microRNA-223 and transcription factors NFI-A and C/EBPalpha regulates human granulopoiesis. *Cell*.

[32] Johnnidis JB, Harris MH, Wheeler RT (2008). Regulation of progenitor cell proliferation and granulocyte function by microRNA-223. *Nature*.

[33] Lu TX, Lim E, Besse JA (2013). MiR-223 deficiency increases eosinophil progenitor proliferation. *Journal of Immunology*.

[34] Li T, Morgan MJ, Choksi S, Zhang Y, Kim Y, Liu Z (2010). MicroRNAs modulate the noncanonical transcription factor NF-*κ*B pathway by regulating expression of the kinase IKK*α* during macrophage differentiation. *Nature Immunology*.

[35] Shi L, Fisslthaler B, Zippel N (2013). MicroRNAs-223 antagonises angiogenesis by targeting *β*1 integrin and preventing growth factor signaling in endothelial cells. *Circulation Research*.

[36] El Gazzar M, McCall CE (2012). MicroRNAs regulatory networks in myeloid lineage development and differentiation: regulators of the regulators. *Immunology and Cell Biology*.

[37] Fukao T, Fukuda Y, Kiga K (2007). An Evolutionarily Conserved Mechanism for MicroRNA-223 Expression Revealed by MicroRNA Gene Profiling. *Cell*.

[38] Mueller BU, Pabst T (2006). C/EBP*α* and the pathophysiology of acute myeloid leukemia. *Current Opinion in Hematology*.

[39] Mendler JH, Maharry K, Radmacher MD (2012). RUNX1 mutations are associated with poor outcome in younger and older patients with cytogenetically normal acute myeloid leukemia and with distinct gene and microRNA expression signatures. *Journal of Clinical Oncology*.

[40] Kuchenbauer F, Mah SM, Heuser M (2011). Comprehensive analysis of mammalian miRNA∗species and their role in myeloid cells. *Blood*.

[41] Feng J, Iwama A, Satake M, Kohu K (2009). MicroRNA-27 enhances differentiation of myeloblasts into granulocytes by post-transcriptionally downregulating Runx1. *The British Journal of Haematology*.

[42] Ben-Ami O, Pencovich N, Lotem J, Levanon D, Groner Y (2009). A regulatory interplay between miR-27a and Runx1 during megakaryopoiesis. *Proceedings of the National Academy of Sciences of the United States of America*.

[43] Min S, Li L, Zhang M (2012). TGF-*β*-associated miR-27a inhibits dendritic cell-mediated differentiation of Th1 and Th17 cells by TAB3, p38 MAPK, MAP2K4 and MAP2K7. *Genes and Immunity*.

[44] Tili E, Croce CM, Michaille JJ (2009). miR-155: on the crosstalk between inflammation and cancer. *International Reviews of Immunology*.

[45] Rodriguez A, Vigorito E, Clare S (2007). Requirement of bic/microRNA-155 for normal immune function. *Science*.

[46] Higgs G, Slack F (2013). The multiple roles of microRNA-155 in oncogenesis. *Journal of Clinical Bioinformatics*.

[47] Ober C, Cox NJ, Abney M (1998). Genome-wide search for asthma susceptibility loci in a founder population. The Collaborative Study on the Genetics of Asthma. *Human Molecular Genetics*.

[48] Taganov KD, Boldin MP, Chang K, Baltimore D (2006). NF-*κ*B-dependent induction of microRNA miR-146, an inhibitor targeted to signaling proteins of innate immune responses. *Proceedings of the National Academy of Sciences of the United States of America*.

[49] Zhao JL, Rao DS, O'Connell RM, Garcia-Flores Y, Baltimore D (2013). MicroRNA-146a acts as a guardian of the quality and longevity of hematopoietic stem cells in mice. *eLife*.

[50] Rusca N, Dehò L, Montagner S (2012). miR-146a and NF-*κ*B1 regulate mast cell survival and T lymphocyte differentiation. *Molecular and Cellular Biology*.

[51] Williams AE, Perry MM, Moschos SA, Larner-Svensson HM, Lindsay MA (2008). Role of miRNA-146a in the regulation of the innate immune response and cancer. *Biochemical Society Transactions*.

[52] Lu TX, Lim E, Itskovich S (2013). Targeted ablation of miR-21 decreases murine eosinophil progenitor cell growth. *PLoS ONE*.

[53] Case SR, Martin RJ, Jiang D, Minor MN, Chu HW (2011). MicroRNA-21 inhibits toll-like receptor 2 agonistinduced lung inflammation in mice. *Experimental Lung Research*.

[54] Vennegaard MT, Bonefeld CM, Hagedorn PH (2012). Allergic contact dermatitis induces upregulation of identical microRNAs in humans and mice. *Contact Dermatitis*.

[55] Lu TX, Sherrill JD, Wen T (2012). MicroRNA signature in patients with eosinophilic esophagitis, reversibility with glucocorticoids, and assessment as disease biomarkers. *The Journal of Allergy and Clinical Immunology*.

[56] Smigielska-Czepiel K, van den Berg A, Jellema P (2013). Dual role of miR-21 in CD^4+^ T-cells: activation-induced miR-21 supports survival of memory T-cells and regulates CCR7 expression in naive T-cells. *PloS ONE*.

[57] Menéndez P, Padilla D, Villarejo P (2013). Prognostic implications of serum microRNA-21 in colorectal cancer. *Journal of Surgical Oncology*.

[58] Wang Y, Gao X, Wei F (2014). Diagnostic and prognostic value of circulating miR-21 for cancer: a systematic review and meta-analysis. *Gene*.

[59] Tili E, Michaille JJ, Wernicke D (2011). Mutator activity induced by microRNA-155 (miR-155) links inflammation and cancer. *Proceedings of the National Academy of Sciences of the United States of America*.

